# Recent Advances of Biosensors for Detection of Multiple Antibiotics

**DOI:** 10.3390/bios13090850

**Published:** 2023-08-26

**Authors:** Ning Lu, Juntao Chen, Zhikang Rao, Boyu Guo, Ying Xu

**Affiliations:** School of Automation, Hangzhou Dianzi University, Hangzhou 310018, China

**Keywords:** simultaneous detection, antibiotics, artificial intelligence, nanomaterials

## Abstract

The abuse of antibiotics has caused a serious threat to human life and health. It is urgent to develop sensors that can detect multiple antibiotics quickly and efficiently. Biosensors are widely used in the field of antibiotic detection because of their high specificity. Advanced artificial intelligence/machine learning algorithms have allowed for remarkable achievements in image analysis and face recognition, but have not yet been widely used in the field of biosensors. Herein, this paper reviews the biosensors that have been widely used in the simultaneous detection of multiple antibiotics based on different detection mechanisms and biorecognition elements in recent years, and compares and analyzes their characteristics and specific applications. In particular, this review summarizes some AI/ML algorithms with excellent performance in the field of antibiotic detection, and which provide a platform for the intelligence of sensors and terminal apps portability. Furthermore, this review gives a short review of biosensors for the detection of multiple antibiotics.

## 1. Introduction

In recent years, food safety incidents have prompted people to rethink issues such as drug residues and illegal additions in food safety monitoring. According to relative statistics, about 3000 people die from foodborne diseases annually in the United States [1]. Among food safety issues, drug abuse and enrichment in the food chain make antibiotic drug residues a key object in food quality monitoring. The excessive intake of antibiotics through food will lead to cancer, reproductive system damage, or teratogenicity [2]. Notably, unsafe food usually contains one or more antibiotics. Due to the additive or synergistic effect, the coexistence of multiple antibiotics can even enhance toxicity and the detection of single components is insufficient [3,4,5]. At present, the main detection method for antibiotics is the instrumental analysis method, which has a high sensitivity. However, due to their high cost and cumbersome pretreatment steps, traditional instrumental analysis methods will no longer quickly satisfy the increasing number of samples, and most of the methods can only be applied to the detection of a single target antibiotic. Therefore, it is necessary to develop rapid, high-throughput, and cost-effective methods for the simultaneous detection of multiple antibiotics to overcome these challenges. Simultaneous detection methods have many outstanding advantages. First, the analysis of multiple targets can be achieved in one detection process, reducing the sample consumption and testing cost. Second, by analyzing multiple substances in one operation, high-throughput analysis can be achieved, reducing labor force and improving detection efficiency. These advantages will meet the market demand for commercial sensors. Up until now, the research directions for detecting multiple antibiotic residues have mainly included: developing methods for the simultaneous detection of multiple antibiotics residues to increase detection speed and reduce detection costs; adopting methods with high sensitivity and confirmation ability to improve detection accuracy and reduce detection limits. Moreover, appropriate data statistics and analysis methods should be applied for the collected sensing data to improve the accuracy of sensing data analysis.

A biosensor is a type of diagnostic device that uses immobilized biological components (enzymes, antigens, antibodies, hormones, etc.) or the organism itself (cells, organelles, tissues, etc.) as sensitive elements. Due to the use of biological materials as sensitive elements, biosensors are highly selective and are an ideal analytical tool for quickly and directly obtaining compositional information of complex systems. The design of biosensors involves many disciplines such as chemistry, physics, biology, nanotechnology, communication systems, etc. [6]. In recent years, in order to meet the need for detection under different complex conditions, various modification elements, such as antibodies, aptamers, nanomaterials, and detection methods have been innovated [7,8,9,10,11]. Because biological materials can increase the specificity and stability of target detection substances, they have provided strong benefits to novel biosensors [11]. The growth of these nano materials, which have ideal large surface-to-volume areas or biocompatibility, can help to achieve better sensitivity and detection efficiency [12]. The merit of artificial intelligence- and machine learning technology-based biosensors lies in that, on one hand, they can be applied to processing large sensing data, reducing the workload of manual processing [13]. On the other hand, they can process sensing signals with a certain amount of noise and interference [14]. In addition, the fusion of AI/ML technology can extract more useful features and efficiently analyze and process data [15].

Therefore, the purpose of this review is to present a timely discussion of and perspectives on advanced technologies and their applications in multi-antibiotic detection. Based on the necessity and urgency of the simultaneous detection of multiple antibiotics proposed above, the biosensors using different biological materials as sensitive elements in antibiotic detection are summarized. In addition, the commonly used sensor types based on different detection mechanisms that can be used for the simultaneous detection of multiple antibiotics were reviewed, and the multidimensional features extracted from the detecting methods of sensors were analyzed. Importantly, novel algorithms suitable for antibiotics analysis are discussed with emphasis. Finally, the application and future development of the above technology in the simultaneous detection of multiple antibiotics are prospected.

## 2. Antibiotic Recognition Elements

There are several main principles for biosensors to recognize antibiotics. One is to use immobilized antibodies as recognition elements. Immobilizing antibodies that specifically bind to antibiotics on the surface of the sensor can directly detect antibiotics [16]. The second principle involves the use of aptamers. RNA and DNA aptamers can bind to target analytes through ionic interactions, Van der Waals forces, or hydrogen bonds for detectable analytes. In addition to the use of aptamers and antibodies as recognition elements for antibiotics, another emerging method is the use of MIPs. Since biological materials are used as the sensitive elements of the sensor, biosensors with highly selective materials are an ideal analytical tool for quickly and directly obtaining information on the composition of complex systems when antibiotics coexist.

### 2.1. Antibody

**Figure 1:**
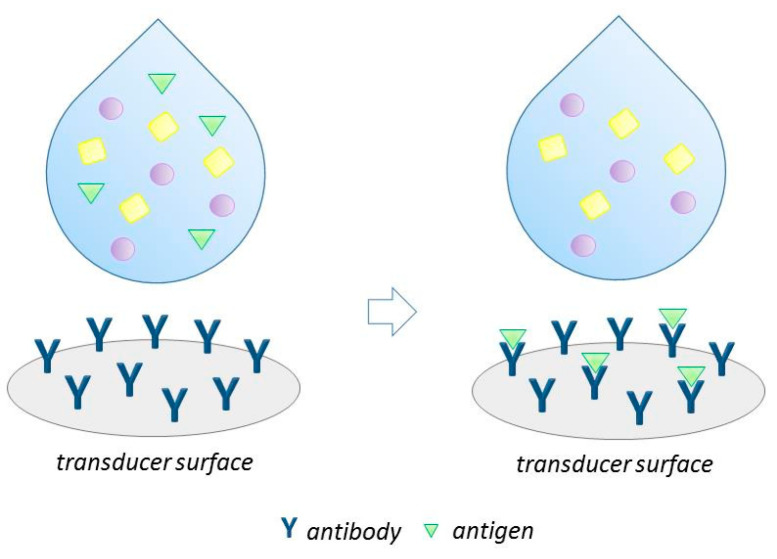
Illustration of immunosensing principle [22]. Graphics with different shapes represent different types of substances in solution, and triangles represent antigens.

Antibodies are commonly used as biorecognition elements in biosensors to develop immunosensors. The principle of immune sensing is illustrated in Figure 1. In the past 40 years, the development of immunosensors has focused on reducing the incubation time, amplifying the detection signals, the synthetizing of functional antibody, and optimizing the antibody immobilization methods. Specific antigen–antibody interactions provide the unique selectivity and high sensitivity of immunosensors, which are considered for their ease of use, simplicity and reliability, short response time, miniaturized application flexibility, and ease of integration into multifunctional analytical sensors [17]. The main principle of biosensors using antibodies as recognition elements for realizing the simultaneous detection of multiple antibiotics is to detect multiple quantum dot or other signal probe materials coupled to different target antibiotic antibodies. For instance, to achieve multi-antibiotic detection in milk, Song et al. [18] realized the simultaneous detection of antibiotics streptomycin (SM), tetracycline (TC), and penicillin G (PC-G). Antibodies (Abs) of antibiotics that were conjugated to quantum dots (QDs) of different emission wavelengths as detection probes (QD-Ab). Then, a direct competitive fluoroimmuno assay was performed based on the fluorescence of the QD-Ab probe, through which the three residue antibiotics were visually and simultaneously determined. The linear ranges for SM, TC, and PC-G were 0.01–25 ng/mL, 0.01–25 ng/mL, and 0.01–10 ng/mL, respectively, with a detection limit of 5 pg/mL for each sample. Compared to similar commercial products, this method can realize the simultaneous detection of multiple target antibiotics, and improve the accuracy, sensitivity, analysis efficiency of three antibiotic residues in milk.

However, the fluorescence detection method is time-consuming and hard to use on-site. LFT is a suitable choice for the rapid and instant determination of antibiotic residues. By designing detection in different areas of the paper, only one signal material is available to achieve multi-target detection. Usually, the signal materials include AuNPs, AgNPs, QDs, and amorphous CNPs [19,20]. Li et al. [21] designed a system with a simple sample preparation strategy to detect antibiotic residue in honey. Relying on QD probes conjugated to the antibiotic monoclonal antibodies, the lateral-flow immunochromatographic assay (LFIA)-based strip and handheld sensor reader can simultaneously detect sulfonamides (SAs) and tetracyclines (TCs) with a high sensitivity, extremely low detecting limits of 0.4 μg/kg, and good specificity. 

The above-mentioned antibody-based immunoassay methods still have defects due to the unstable chemical properties of antibodies, easy changes in antibodies during synthesis, and alteration between antibody batches in the synthesis process. A promising improvement method is to improve the synthesis process of antibodies or to find alternative biorecognition receptors.

### 2.2. Aptamers

**Figure 2:**
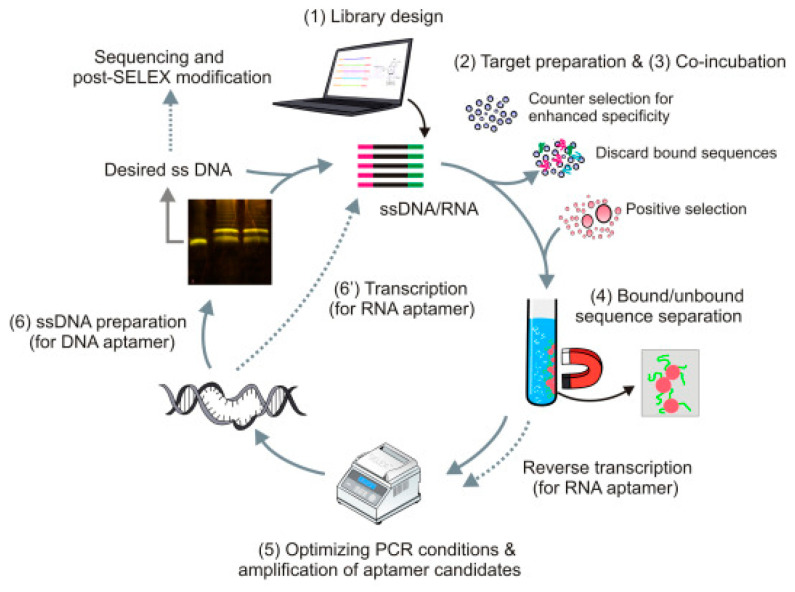
Important steps of a typical SELEX protocol. Reproduced from reference [25] with permission. Copyright 2019, Elsevier.

As a powerful alternative to antibodies, aptamers have good thermal stability, less batch variation, low immunogenicity, and a low cost [23]. Aptamer biosensors use aptamers as recognition components to convert target signals into measurable sensing signals. They can be easily labelled with multiple nanomaterials or redox probes to construct multi-analyte aptasensors with high specificity and the on-spot detection of complex matrices [24]. Aptamers are synthetic oligonucleotides (DNA and RNA) with a length of 35−100 nucleotides, and are selected through systematic evolution by exponential enrichment in vitro (SELEX). The Figure 2 below shows the important steps of a typical SELEX program.

Due to the high sensitivity and scalability of electrochemical detection systems, as well as the specificity of aptamers, electrochemical aptamers are considered to be efficient devices for the simultaneous detection of multiple target analytes [26]. According to previous reports, a variety of aptamers with high antibiotic specificity have been screened and used to detect multiple antibiotics at the same time. For example, Zhu et al. [26] reported a dual ratio electrochemical aptamer sensor based on carbon nanohorns/anthraquinone-2-carboxylic acid/Au nanoparticles (CNHs/AQ/AuNPs) for the simultaneous detection of malathion (MAL) and omethoate (OMT) in fruit samples. The biosensing system exhibited a linear range from 3 pg/mL to 3 ng/mL for MAL and from 10 pg/mL to 10 ng/mL for OMT. To detect the multiple mycotoxins in vegetable samples simultaneously, Zhu et al. [27] also reported a hairpin DNA-assisted dual-ratiometric electrochemical aptasensor with a high reliability and anti-interference ability for the simultaneous detection of aflatoxin B1 and ochratoxin A. Their work has presented a novel way to fabricate a high-performance aptasensor with a detection range of 10–3000 pg/mL for aflatoxin B1 and 30–10,000 pg/mL for ochratoxin A, respectively.

Although the cost of designing aptamers is lower than that of antibodies, similar to immunosensors, aptamer sensors also face unstable performance during point-of-care testing (POCT) detection because the biological activities will be easily affected by the environment. In addition, so far, only relatively few aptamers can meet the requirements for both selectivity and specificity, which limits the development of multiplex aptasensors.

### 2.3. Molecularly Imprinted Polymers

**Figure 3:**
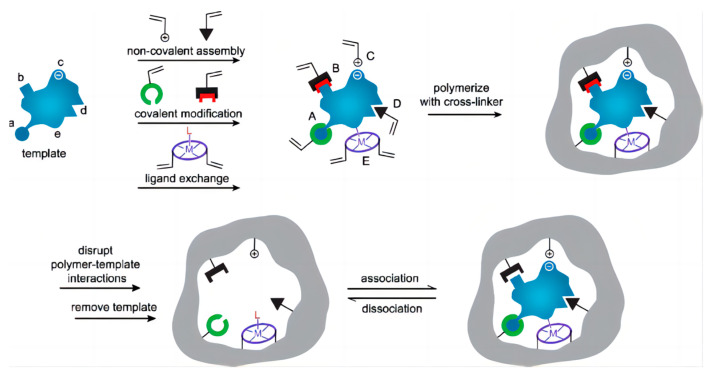
Highly schematic representation of the molecular imprinting process: The formation of reversible interactions between the template and polymerizable functionality may involve one or more of the following interactions: [(A) reversible covalent bond(s), (B) covalently attached polymerizable binding groups that are activated for non-covalent interaction by template cleavage, (C) electrostatic interactions, (D) hydrophobic or van der Waals interactions or (E) coordination with a metal centre; each formed with complementary functional groups or structural elements of the template, (a-e) respectively]. A subsequent polymerization in the presence of crosslinker(s), a crosslinking reaction or other process, results in the formation of an insoluble matrix (which itself can contribute to recognition through steric, van der Waals and even electrostatic interactions) in which the template sites reside. Template is then removed from the polymer through disruption of polymer-template interactions, and extraction from the matrix. The template, or analogues thereof, may then be selectively rebound by the polymer in the sites vacated by template, the ‘imprints’. While the representation here is specific to vinyl polymerization, the same basic scheme can equally be applied to sol-gel, polycondensation etc. Reproduced from reference [29] with permission. Copyright 2006, Wiley Online Library.

A new technology is based on molecularly imprinted polymers (MIPs) whose physical and chemical properties are similar to antibodies. Composite materials are used which are composed of functional monomers, crosslinkers, and template molecules that can withstand a more comprehensive range of pH and temperature than biological materials. Figure 3 illustrates the preparation process of MIPs. This technology has emerged as a promising approach to improve the target selectivity of chemical/biosensors, and has been widely used in the preparation of biosensors for antibiotics [28].

**Figure 4:**
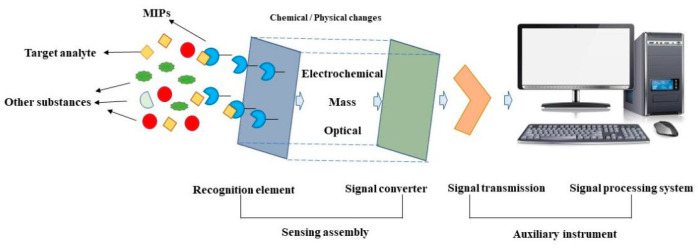
Classic structure and principle of MIP-based sensors. Reproduced from reference [25] with permission. Copyright 2019, Elsevier.

MIP-electrochemical sensors mainly use the interaction between the analyte and the electrode surface to convert the analyzing signal. The current, voltage, etc., will be affected by this effect. The classic structure and principle of MIP-based sensors is illustrated in Figure 4. MIP-based sensors have the advantage of compatibility between MIPs and electrochemical analysis, demonstrating a satisfactory reproducibility, sensitivity, and chemical stability of the template molecules. Since the molecular-specific recognition ability of MIPs is based on template-imprinted sites, it is helpful for the recognition needs of various molecules in biological analysis [30]. In the detection and analysis of antibiotics, MIP-based electrochemical sensors are important methods for the analysis of antibiotic residues in complex matrices. The molecule-specific recognition capability of MIPs is based on template-imprinted sites, which contribute to a variety of recognition events in biological analyses.

To track and quantify trace amounts of drugs in biological tissue samples, Liu et al. [31] proposed an anti-matrix interference, highly sensitive, and highly reproducible multiple-template-imprinted polymer (MMIP) for the monitoring of trace sulfa antibiotics (SAs) in mouse tissue samples. Based on theoretical calculation and experimental optimization, the molar ratios of sulfamethoxazine (SMA), sulfamethoxazole (SMO), and sulfachloropyrazine (SCPA) to MMIP were estimated by the UHPLC-MS/MS method. The ultra-sensitive monitoring and multi-component quantitative measurement of sulfonamide antibiotics in samples were realized. The method showed an excellent linearity of detection in the range of 0.1–500 μg/L, and the ultrasensitivity the with lowest limits of detection was 0.03 μg/L. The maximum SA residue recovered from sample tissues was 5.48 μg/g. Furthermore, compared with other materials, the MMIPs were reusable. After more than 100 tests, there was only a slight degradation in performance.

Therefore, MIPs are an ideal material that can eliminate sample matrix interference and effectively enrich trace antibiotics [32]. As recognition elements, the detection performance of sensors can be improved. However, increasing the binding ability of MIPs and preventing template leakage are still challenges in designing new MIPs.

## 3. Simultaneous Detection of Multiple Antibiotics Based on Different Methods

The methods for the simultaneous detection of antibiotic residues mainly include the microbial method, electrochemical method, high-performance liquid chromatography and liquid-mass spectrometry, fluorescence method, Raman spectroscopy, etc. Different detection methods have their own advantages and disadvantages. There are also many defects in the development of methods for the simultaneous detection of multiple antibiotics. First, cross-reactivity cannot be neglected when the structure of the target analytes is similar. Second, multi-target detection requires different signal labels, and signal overlap and mutual interference are not conducive to detection and analysis. Third, complexity of the sample matrix will affect the detection results. In the past few years, more and more related studies have tried to overcome the above-mentioned problems [33,34,35,36]. The following section will introduce these sensors in detail. Nanomaterials have the characteristics of surface effect, quantum effect, and small size effect. They exhibit a series of unique optics, magnetism, electricity, good biocompatibility, and catalytic ability when combined with these sensing methods, and have been widely used in the field of antibiotic detection.

### 3.1. Fluorescence Method

Fluorescent sensors have attracted considerable attention in the field of the multiplex detection of antibiotics due to their ease of manipulation, fast response, high sensitivity, and potential for instant detection. Among fluorescence methods, multiplex sensors based on Forster resonance energy transfer (FRET) have shown superior performance in the simultaneous detection of a few antibiotics [24]. Fluorescent sensors for the detection of multiple antibiotics usually require the construction of two or more distinguishable fluorescent signals derived from labels on different biorecognition probes to identify target molecules. According to the different presentation forms of the signal, FRET biosensors can be further divided into fluorescence “turn-on”, “turn-off”, ratiometric biosensors, and fluorescence upconversion biosensors. Fluorescent “turn-on” and fluorescent “turn-off” sensors usually utilize fluorophores or quantum dots as fluorescent probes, and the intensity of the fluorescent signal will vary with the presence of the analyte [37]. Due to the superior optical properties of quantum dots, such as a long fluorescence lifetime, photostability, large Stokes shift, low background noise, and large molar extinction coefficient, quantum dots have been favored and widely used in multiplex antibiotic detection [38]. In addition, quantum dots usually have a wide excitation spectrum and a narrow emission spectrum, which can reduce the problem of the mutual interference of different fluorescent signals that often occurs in multiplex detection.

To detect antibiotic residues in chicken meat, Li et al. [39] combined fluorescent nanobiosensors with homemade fluorescence analyzers for the simultaneous identification and quantification of three antibiotics. The mechanism of the system is based on the targeted antibiotics and signal labels of antigen-quantum dots (IQDs) that are competitively attached to the recognition elements of antibody magnetic beads (IMBs) to form conjugates. Then, three quantum dots with different excitation wavelengths are used to divide the three target antibiotics simultaneously. Through magnetic separation, the fluorescent signal in the supernatant is proportional to the concentration of the antibiotic, and the concentration of the antibiotic can be indirectly quantitatively detected by analyzing the fluorescent signal. The fluorescence biosensing system can be used to analyze enrofloxacin, tilmicosin, and florfenicol in chicken samples with detection limits of 0.34 μg/kg, 0.7 μg/kg, and 0.16 μg/kg, respectively.

Since the detection signal of antibiotic small molecules is usually too low to be detected, signal amplification strategy is another active area of research in this field, and the amplification part based on nanomaterials and DNA has attracted continuous attention [40,41].

### 3.2. Electrochemical Method

**Figure 5:**
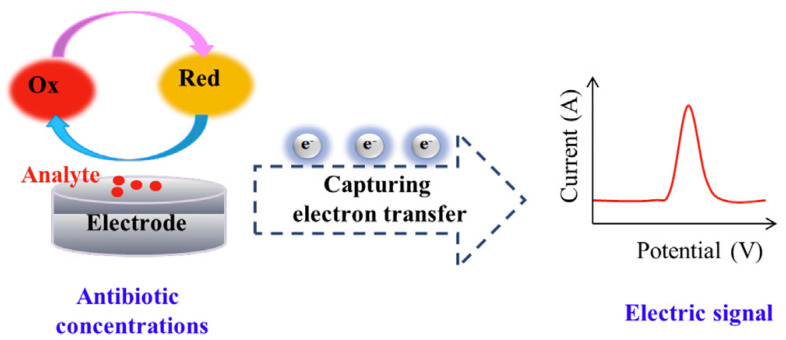
Principle of electrochemical sensor (electrode) for detecting antibiotics. The red line is the spectrum of the electrical signal DPV. Reproduced from reference [42] with permission. Copyright 2021, Elsevier.

An electrochemical sensor is a device for the qualitative or quantitative analysis of target substances. Its essence is that the sensing signal generated by the reaction of the measured substance with a specific sensing element will be converted into an identifiable electrical signal that is proportional to the concentration of the target substance through a specific sensor. Figure 5 shows the basic principle of an electrochemical sensor.

Electrochemical methods can be generally divided into potentiometric methods, amperometric methods, conductometric methods, impedance methods, and voltammetric methods [43]. Among them, the impedance method, differential pulse voltammetry, and ECL are widely used to detect various antibiotics in food [44,45,46,47,48]. For the electrochemical detection of multiplex antibiotics, two or more distinguishable electrochemical signals need to be generated. From existing studies, common materials used to develop distinguishable electrochemical signals are quantum dots, metal ions, Methylene Blue, Ferrocene, etc.

In order to detect multiple antibiotics in milk, based on aptamers and quantum dots, PbS, CdS, and ZnS, Xue et al. [49] designed a sensing system that can simultaneously detect streptomycin, chloramphenicol, and tetracycline. The low detection limits for streptomycin, chloramphenicol, and tetracycline reached 10 nM, 5 nM, and 20 nM, respectively.

In addition, how to improve the sensitivity of multi-channel electrochemical sensors is a key focus for researchers when designing sensors. There are two basic methods to improve the detection sensitivity of electrochemical multi-channel adaptive sensors. The first approach is the electrode modification method based on nanomaterials (carbon nanotubes, graphene nanosheets, etc. [50,51]). Since nanomaterials can improve the electrocatalytic activity or the characteristics of electron transfer reactions, they are beneficial in improving the performance of multiple electrochemical reactions [52,53]. The second method is to amplify electrochemical signals by loading more electrochemical signal labels inside or on the surface of nanomaterials, including AuNPs, hollow silica nanoparticles, MOFs, apoferritin, etc. [54,55,56,57,58,59,60,61]

Taking fish and milk as the detection substances, Shen et al. [61] prepared the coded probes by loading deferrin with Cd^2+^ and Pb^2+^ ions and applied double-stranded DNA markers. By the square wave voltammetry detection method, the peak currents of the labeled Cd^2+^ and Pb^2+^ corresponding to the concentration of kanamycin and ampicillin can realize the simultaneous detection of the two antibiotics within a range from 0.05 pM to 50 nM.

### 3.3. Surface-Enhanced Raman Scattering (SERS) Method

SERS is an ultrasensitive vibrational spectroscopy technique that enhances the vibrational spectroscopy of molecules that are adsorbed on or near metallic nanostructures or surfaces [62,63]. Due to its high sensitivity, specificity, and non-destructive detection, it has been widely used in the detection of food contaminants. SERS can be used to simultaneously detect multiple targets with unique fingerprints in the Raman spectrum [64]. According to the signal enhancement mechanism of SERS, the selection of suitable substrates plays a crucial role in the sensitive detection of various antibiotics. In the past few years, many nanomaterials have been used to develop SERS substrates or labels. For example, Wang et al. [65] prepared a gecko-inspired nano-tentacle SERS substrate for the direct detection of three different antibiotics. The nano-tentacle arrays containing AuNPs and AgNPs were modified by the seed deposition method. The multiple components of the sample were rapidly and reliably determined. In particular, under the optimal conditions, a sensitivity of 1.6 ng/cm^2^ (S/N = 3) for thiram was obtained on apple peels with a correlation coefficient of 0.99.

When using the SERS method to monitor antibiotic residues in food, methods such as liquid–liquid extraction and solid-phase extraction are commonly used to preprocess the sample to extract analytes from complex food samples. However, these methods are cumbersome, laborious, time-consuming, and not environmentally friendly, which limits the online analysis application of SERS technology. Jin et al. [66] proposed a simple and sensitive hollow cellulose microextraction combined with SERS (HF-LPME-SERS) technology for the rapid detection of multiple antibiotics in egg samples. In the HF-LPME system, the target analytes are extracted from the water sample into the organic phase in the hollow fiber hole, and then further into the absorption phase inside the hollow fiber lumen. This method is superior to the traditional extraction method with simpler operation and a lower cost, and can thus significantly reduce the amount of organic solvent used. In addition, a laser-induced self-assembled SERS chip can further directly extract the measurement spectrum. The results showed that 11 antibiotics in eggs could be tested rapidly and economically by the novel HF-LPME-SERS method, and the concentrations of multiple antibiotics in egg were detected as low as 10 ng/g with a RSD of 23.4%.

### 3.4. Colorimetric Method

The key principle of the colorimetric method is to analyze the color changes in the analyte for detection by the naked eye or portable, low-cost equipment instead of other more complicated equipment, and the output signal is directly visualized [67,68,69]. Metallic nanoparticles have received widespread attention in colorimetric sensors due to their optical properties related to distance/size [70]. Antibodies and aptamers can be easily adsorbed on the surface of metallic nanoparticles with stronger affinity through non-potential electrostatic response [71]. For multi-antibiotic detection, they can be detected by visually reading and measuring the state changes in the solution using UV-vis spectroscopy [72,73,74,75]. In particular, AuNP-based colorimetric sensors have attracted great interest for detecting antibiotic residues in food due to their extremely high optical extinction coefficient and unique properties [76,77].

Aptamers are ideal biomolecules and sensing elements for colorimetric aptasensors. The key point to be solved in establishing AuNP-based colorimetric aptasensors is the aggregation and flocculation of unmodified AuNPs, which are induced by the passivating surface layer, ionic strength, pH, and temperature. A feasible method is to covalently link thiol groups of ssDNA on gold nanoparticles [78]. For example, in order to detect tetracycline and chloramphenicol in milk, Wu et al. [79] designed a multifunctional adapter, which can be adsorbed on the surface of AuNPs. For the two antibiotics, due to the differentiated recognition group with different lengths of the corresponding aptamers, distinctive observed colors can be quantitatively detected by UV–vis spectroscopy. The reported sensor exhibited remarkable sensitivity for the detection of chloramphenicol and tetracycline, with LODs of 7.0 and 32.9 nM, respectively.

However, since each method has its unique characteristics, so far, there is no optimal method for multiple antibiotic detection. Generally speaking, fluorescence, colorimetric, and electrochemical sensors are the common choice for high sensitivity and rapid detection, while SERS-based sensors are an ideal choice for the non-destructive detection of antibiotics in food because of their non-invasive detection. The above-mentioned types of sensors for multiplex antibiotic detection have shown a superior detection performance under the optimal conditions, but they are not suitable for on-site detection due to the large-scale equipment that is required. Based on the lateral-flow test strips (LFT), the microfluidic chip can meet the requirements of on-spot detection and is the development direction of future sensors due to it having the characteristics of miniaturization, portability, and easy-to-use analysis.

## 4. Artificial Intelligence/Machine Learning Algorithms for Antibiotic Detection

Nowadays, artificial intelligence and machine learning technology (AI/ML) have allowed for impressive progress. Some innovative algorithms have been successfully applied in many fields like image processing, face recognition, natural language processing, and medical data [80,81,82,83,84,85]. However, for biosensors, especially in the field of multi-antibiotic detection, there are still relatively elusive situations. The following will be a discussion based on multi-antibiotic detection methods.

### 4.1. Benefits in Biosensing Antibiotics by AI/ML Algorithms

In the process of traditional empirical model processing and the optimization of experimental methods for antibiotic detection, it is necessary to analyze multiple independent factor variables. Since only one variable is changed while all other variables are kept unchanged [86], this method obviously requires a lot of data analysis. The traditional experiment is time-consuming and labor-intensive. In addition, aging biosensors have insufficient repeatability and exhibit stability deterioration in actual sample detection. There are a large number of interfering substances in real samples, as well as differences in temperature, pH conditions, and laboratory environments. These problems are important bottlenecks that hinder the commercialization of biosensors. Therefore, artificial intelligence technology coupled with accelerated biosensors is another way to optimize sensors to improve immediacy, accuracy, and reliability in real sample measurement sensors.

One of the applications of artificial intelligence in sensor design is to efficiently process complex matrices or large-sample sensor detection data. By selecting appropriate machine learning algorithms, the hidden relationship between the sample parameters and sensor signals can be discovered through data visualization and mine the relationship between the signal and the target detection samples. Traditional sensor data processing often applies a single feature of the collected data as an indicator of the concentration of the detected sample, and the relationship between them is established. However, one-dimensional data analysis is not enough to obtain a sensitive signal that is highly correlated with the type and quantity of the analyte, and it is necessary to combine multiple independent factor variables with artificial intelligence tools. 

Moreover, the complexity and multivariate analysis of biological systems and environments is another challenge for the current high-throughput sensing methods and multi-analyte identification design. For example, due to the reactions between substances when performing cyclic voltammetry testing of multi-materials, there are several types of curves can be observed, such as separate current peaks, the coexistence of independent peaks and coupled peaks, and several peaks merged into one peak due to coupling reactions [87]. Especially, when the molecular volume of and orbital differences in molecular substances are small, the overlapping of current signals cannot satisfy the detection and identification of multiple analytes. Therefore, artificial intelligence methods should be combined to decouple and overcome this challenge for the detection of antibiotics [88,89,90,91,92]. 

### 4.2. General Process and Principle of Data Analysis

**Figure 6:**
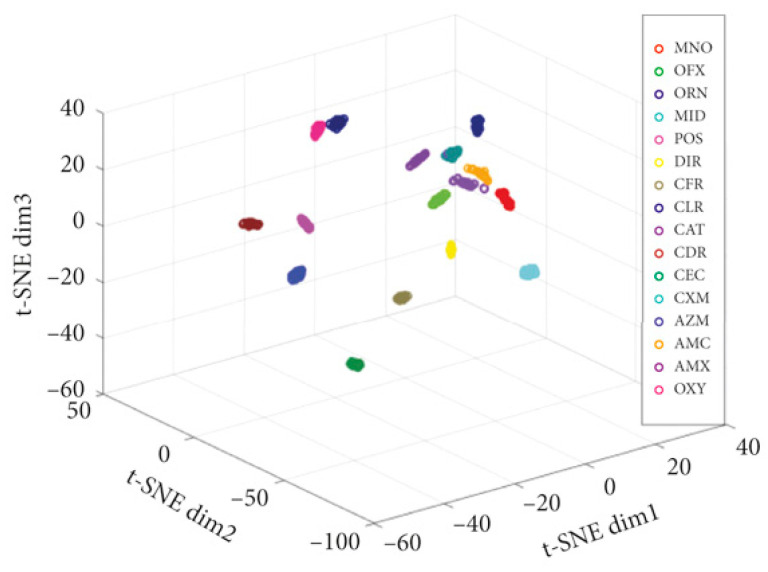
The visualization plots of different types of antibiotics using t-SNE [94].

**Figure 7:**
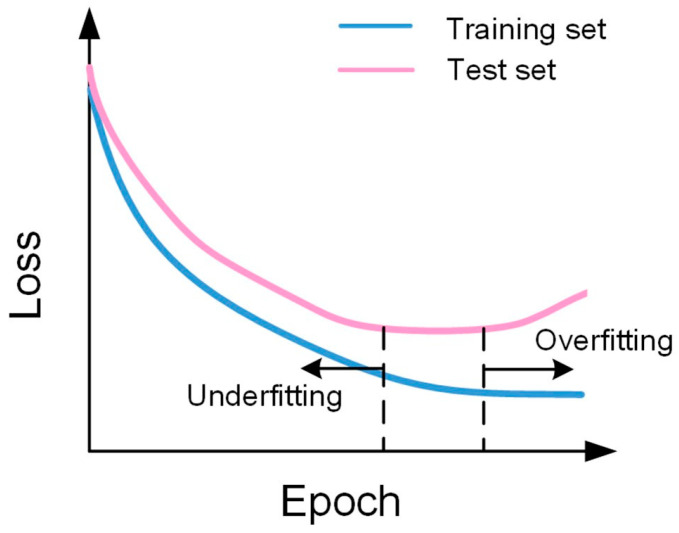
Loss curve in the training stage. Reproduced from reference [95] with permission. Copyright 2019, Elsevier.

Designing an appropriate AI/ML model according to the type of data set and analysis purpose (qualitative or quantitative detection) will help us to extend detection methods to different data acquisition devices and application scenarios. To design an AI/ML model for a specific biosensing system, the following details in the process need to be focused: (1) the size of the input data set and the dimension of the input value. At present, biosensing data mainly include sequence data sets obtained from electrochemical and spectral biosensors or image data sets acquired by colorimetric and fluorometric biosensors. (2) The feature selection and feature extraction processes: through feature selection, the prediction accuracy of the model, computational cost, risk of overfitting, and interpretability of data can be improved. It is necessary to choose an appropriate feature selection method according to the specific situation to avoid the influence of too many features on the model. The main methods of feature selection include the filter, wrapper, and embedded methods. The main methods of feature extraction include principal component analysis, linear discriminant analysis (for sequence data), and the scale-invariant feature transform method (for image data). (3) The pre-processing and post-processing according to the application scenarios of the AI/ML model: general preprocessing methods include derivatives, denoising, Fourier transform, etc. The different preprocessing methods are applicable to data from different sources. For Raman spectra, each spectrum requires Savitzky-Golay smoothing, background subtraction, and min-max to [0,1] [93]. Post-processing refers to the process of processing data after data analysis. Post-processing should include operations such as data visualization, data mining, and data modeling. The purpose of post-processing is to better interpret the data, so as to make reasonable data analysis and decision-making. As shown in Figure 6, t-Stochastic Neighbor Embedding(t-SNE) is a nonlinear dimensionality reduction algorithm for mining high-dimensional data, which can map multi-dimensional data into two-dimensional or three-dimensional space. Therefore, t-SNE is very suitable for visualization operations on high-dimensional data. (4) The model architecture, including number of layers, activation functions, loss functions, and the data dimension of the entire processing pipeline: the loss curve is a key indicator for reporting the training status. It can also reflect varying degrees of influence of hyperparameters. Figure 7 exhibits a classic loss curve showing both overfitting and underfitting. (5) The model development, including model training and tuning processes, such as transfer learning, regularization methods, ensemble methods, tuning thresholds, performance evaluation indicators, etc.: hyperparameter tuning is a key task in the analysis of sensory data in the verification phase. The parameters of the algorithm include the number of hidden neurons, learning rate, batch size, etc. The classic cross-validation method can be applied to hyperparameter tuning and evaluate the predictive performance of the algorithm after parameter tuning. As for metrics, in the field of biosensing, the correlation coefficient (R^2^), the relative error of prediction (REP), and root-mean-square difference (RMSD) can be adopted to evaluate the performance of the model.

### 4.3. Various Algorithms of AI/ML and the Application in Antibiotic Detection 

**Figure 8:**
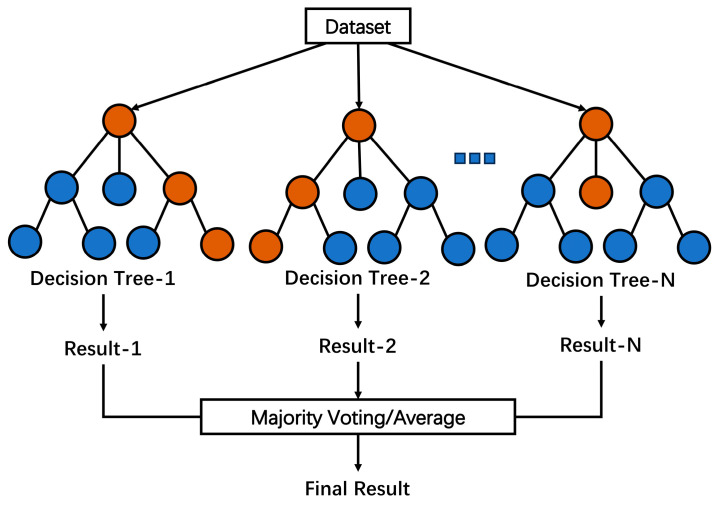
Schematic structure of the RF algorithm.

Random Forest (RF): As shown in Figure 8, RF is an ensemble learning method that combines multiple decision trees to improve the performance and robustness of the model [96]. The core of the random forest algorithm includes random sampling, random feature selection decision tree construction, and integrated decision-making. At each node of the decision tree, a random subset of features is considered for splitting. This helps to introduce randomness and reduce correlation between trees. Overfitting is avoided by randomly selecting a portion of samples and features for training when building each decision tree. In the process of obtaining an integrated decision, the prediction results are considered comprehensively. For classification tasks, the class prediction is determined by majority voting among the decision trees. For regression tasks, the output is the average prediction of all of the trees. A successful application of the RF algorithm was the construction of a dual emission fluorescence/colorimetric sensor for the determination of nine antibiotics by Xu et al. [97]. Detection data from the array sensor were processed and analyzed by the extreme random forest algorithm, which is an ensemble of unpruned classification or regression trees. In addition, the classification accuracy of unknown samples outside the data set was 100%, and the average concentration error was less than 5%.

**Figure 9:**
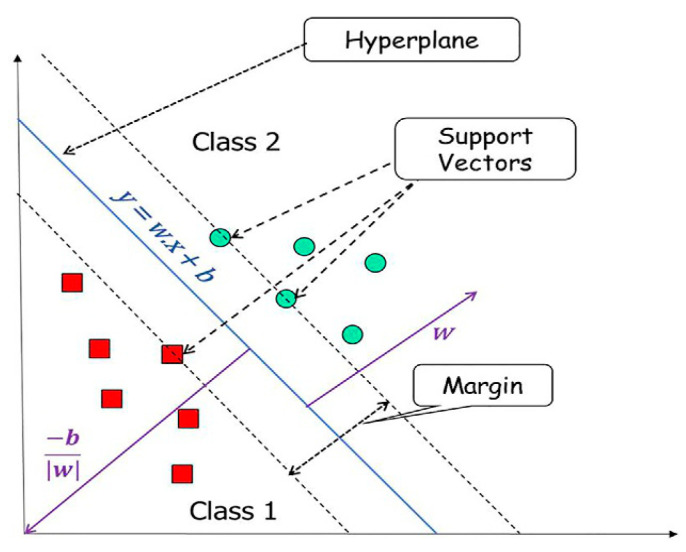
Components of SVM. Graphics with different shapes represent different class of data. Reproduced from reference [98] with permission. Copyright 2022, Elsevier.

Support Vector Machine (SVM): SVM is a supervised learning ML algorithm used for classification and regression tasks. The principle of the algorithm is to find a hyperplane in the feature space that can effectively separate samples of different categories and achieve the largest interval (Margin). Figure 9 depicts the key components of the SVM algorithm. For nonlinear problems, the SVM algorithm can map the data to a higher-dimensional space through the kernel function, so as to find a linearly separable hyperplane in the new space. The SVM algorithm performs well on small samples and high-dimensional data, which can handle complex decision boundaries. Guo et al. [94] reported a t-SNE-PSO-SVM algorithm to analyze the terahertz spectral data of 16 antibiotics to solve the problem that antibiotics with the same absorption peak could not be distinguished by spectral features. In order to reduce the training time, t-SNE was used to reduce the dimensionality of the absorption spectrum data with a dimension of up to 143, and the 3D PCA of the absorption spectra of different antibiotics was displayed by the t-SNE algorithm. After dimension reduction, the new data matrix was trained by the SVM model and the parameter was optimized with the particle swarm optimization (PSO) algorithm. The prediction accuracy of the model is 99.91%, which is an ideal method for antibiotic identification.

**Figure 10:**
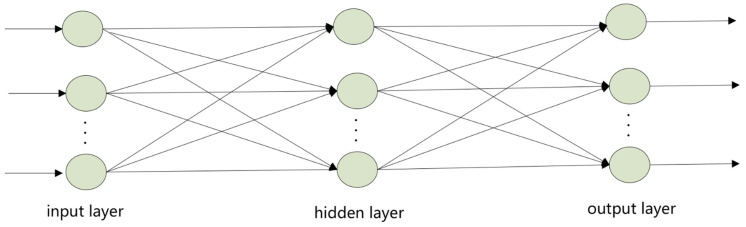
Structure diagram of BP-ANN [103].

Artificial Neural Networks (ANN): ANN are composed of multiple artificial neurons (nodes) and connections between neurons, and a layered artificial neural network generally includes input, output, and multiple hidden layers. The nodes process the given signals and transmit them to the next connected node. The output of a node depends on the weighted sum given by the nodes in the previous layer. The key parameter that significantly affects the performance of artificial neural networks is the size of the hidden layers [99]. Containing only one hidden layer, the MLP network model trained by the backpropagation (BP) algorithm is widely used in artificial neural networks. BP-ANN is a quite representative and widely used model in MLP networks [100,101]. The classic structure of the BP-ANN feedforward network is shown in Figure 10. Shawkat et al. [102] made quantitative measurements of three types of bacteria based on changes in impedance under the same measuring conditions. The real-time impedance changes in the three types of bacteria were measured by the impedance analyzer and the data were treated as input for classification. Three targets of the same concentration (10^6^ CFU) can be quickly detected and classified by analyzing the impedance test data after 8 min. Therefore, the BP-ANN method was applied to classify these bacteria. The key features included the measuring power, the I-V curve, and the first derivative and second derivative of I-V characteristics, and the classification accuracy reached almost 100%. Therefore, it can be applied as an early detection and classification tool in the food industry.

**Figure 11:**
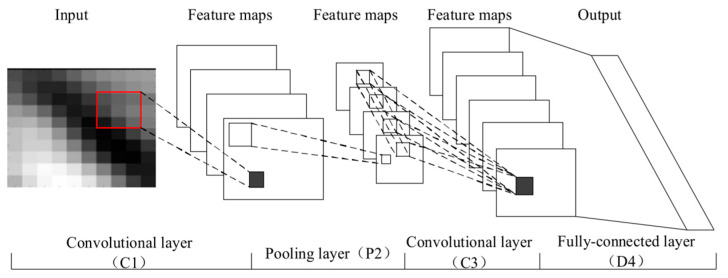
Structure of Convolutional Neural Network Structure [104].

Convolutional Neural Network (CNN): The core parts of CNNs include the convolution layer, pooling layer, convolution kernel, activation function, and fully connected layer (Figure 11). As the core component of the convolutional neural network, the convolutional layer acts as an image extraction function. In a complex neural network, there are generally multiple convolutional layers with convolution kernels of different sizes and designs to set the step size in the image and traverse to extract image features. The fully connected layer is the outlet of the model results, which adapt to different fully connected layers and realize the transformation of the model classification or regression tasks. In addition to using traditional machine learning indicators to evaluate the quality of the model, CNNs also introduces the Cross-Entropy Loss function (Cross-Entropy Loss) to evaluate the model training effect, which effectively shows the difference between the prediction result and the real label. In addition, the visualization of network layers also provides a way to understand the specific characteristics of the model. Therefore, in order to process high-throughput visualized spectral data, Huang et al. [91] used the CNN based on the GoogLeNet framework to establish the relationship between image features and antibiotic concentrations. The dataset with sufficient chromogenic features was obtained by using a scheme of multispectral and mixed chromogenic systems together. The results show that the method had a good detection effect for the three target antibiotics that were detected, and the average relative error was about 5% in the concentration range of 200–800 mg/L. 

According to the information mentioned above, AI/ML algorithms can help to discover unknown complex relationships between similar data with a high inclusivity for the dimensionality of the data source. It can simplify the process of analyzing experiment results and improve the interpretation of complex sensing systems such as the detection of the coexistence of multiple antibiotics. Therefore, it is foreseen that AI/ML will be widely used in multi-antibiotic detection. However, in actual scientific research work, there are two unavoidable challenges. One is to find an efficient and suitable AI/ML algorithm for a specific biosensing system, and the other is how to provide a large number of training data for the established algorithm model in a short experimental time. Although many successful cases have proven that the AI/ML algorithm has excellent advantages in substance identification, up until now, there is still no suitable method that can effectively collect enough antibiotic sensing data sets. A promising solution is to find suitable sensing strategies coupled with AI/ML to effectively drive forward the development of technology for the detection of an environment. Furthermore, for quantitative detection, the algorithm has a satisfactory predictive effect only in a limited range, and higher precision algorithms still need to be developed. As for biosensors integrated with AI/ML algorithms, there are different statistical methods for performance indicators. However, each unique AI/ML algorithm has its own most-valuable types of input data, and unified evaluation indicators and methods are needed. Also, it is necessary to control information security during data collection, transmission, and the processing of sensor development.

## 5. Conclusions and Outlook

At present, the efficient and rapid detection of multi-antibiotics is of great significance. However, the main challenges are the selection of front-end modifiers and the rapid and accurate result output of the back end. This review introduces the current detection techniques, biometric probes, and nanomaterials that are commonly applied in multi-antibiotic detection. In particular, the application of AI/ML algorithms in multi-antibiotic detection is reviewed, and the impact of this emerging technology on multi-antibiotic detection is illustrated. Although the developed multi-antibiotic detection sensors have achieved satisfactory results, there are still limits in the laboratory testing environment and the operation of professionals, and there are still many challenges in practical applications. These challenges include the need to fix different biological receptors that are applied to constructing biosensor systems for ensuring the biocompatibility of materials and biometric molecules; the influence of non-uniformity of nanoparticles on the performance of biosensors; cross interference within antibiotics; and the effective connection between nanomaterials and biological recognition molecules.

The detection of antibiotics in food is relatively mature. In the previous chapters, many methods for the detection of antibiotics in foods such as milk, honey, and eggs have been discussed. However, antibiotic contamination may occur in various situations, especially in aquatic environments, which is continuously harmful to human health. The pollution sources of antibiotics in water mainly include wastewater discharged from domestic sewage, medical wastewater, animal physiology, and aquaculture. Up until now, there are only a few methods can simultaneously be adopted to detect multiple antibiotics in water samples [105,106,107]. The focus of these studies is mainly on developing new antibiotic extraction techniques, synthesizing new separation techniques, and improving water quality through the solid-phase extraction of MIP materials. For the above-mentioned requirements of various detection scenes, in addition to designing high-performance biosensors that can cope with various detection environments, it is quite necessary to integrate AI/ML algorithms. In the process of applying AI/ML algorithms, the most important factors are the feature extraction process based on the detecting method of the antibiotic, and the selection of appropriate AI/ML methods to reduce analysis errors. At the same time, it is very important to apply interpretable AI/ML models to POCT electronic devices for multi-antibiotic detection.

## Data Availability

Not applicable.
